# Are they eosinophil extracellular traps?

**DOI:** 10.3389/fimmu.2025.1686316

**Published:** 2025-12-17

**Authors:** Pedro Chacón, Adrián López-Postigo, Javier Monteseirín, David Ribas-Pérez, Antonio Vega-Rioja

**Affiliations:** 1 Laboratorio de Inmunología y Alergia-FISEVI, UGC de Alergología, Hospital Universitario Virgen Macarena, Sevilla, Spain; 2 Departamento de Medicina, Facultad de Medicina, Universidad de Sevilla, Sevilla, Spain; 3 Hospital Quirón Sagrado Corazón, Hospital Quirón Infanta Luisa, Sevilla, Spain; 4 Facultad de Odontología, Universidad de Sevilla, Sevilla, Spain

**Keywords:** asthma, neutrophil extracellular traps (NETs), allergy, eosinophils, eosinophil extracellular traps (EETs)

## Abstract

This perspective critically evaluates the reliance on eosinophil cationic protein (ECP) as a marker for eosinophils in identifying extracellular traps (ETs) in asthma research, as exemplified in the study by Lu et al. While traditionally associated with eosinophilic activity, ECP is also produced by neutrophils, monocytes, and other myeloid cells, undermining its specificity. This limitation risks misattributing ET origin, leading to incorrect pathophysiological interpretations and misdirected therapeutic strategies. In allergic asthma, our findings demonstrate that sensitized neutrophils—rather than eosinophils—form ECP-positive ETs in response to relevant antigens, challenging conclusions that ECP^+^/cit-H3^+^ structures necessarily represent eosinophil extracellular traps (EETs). We advocate for the use of more specific eosinophil markers, such as eosinophil peroxidase (EPO) or major basic protein (MBP), in combination with neutrophil-specific markers, alongside rigorous methodological controls and clinically relevant human models. Accurate identification of ET cellular origins is essential for understanding asthma’s heterogeneous inflammatory mechanisms and for guiding the development of targeted, phenotype-specific therapies.

## The importance of extracellular traps in asthma research

Extracellular traps (ETs) function to entrap and immobilize pathogens, thereby contributing to host defense and infection neutralization (1). However, ETs also play a critical role in the pathogenesis of asthma by disrupting bronchial epithelial integrity, amplifying inflammatory signaling, and exacerbating airway dysfunction (2, 3). Notably, elevated ET levels in airway or blood samples have been correlated with disease severity (4).

Despite their increasing mechanistic relevance, ETs have not yet been adopted in clinical practice as biomarkers, largely due to limited standardization and insufficient methodological consensus (5). Nevertheless, growing evidence suggests that understanding ET biology and modulating their formation could offer promising avenues for therapeutic intervention and serve as potential indicators of asthma severity (5–10). Some therapeutic approaches are being explored in the context of other inflammatory diseases, including PAD4 inhibition for cystic fibrosis and DNase therapy to aid recovery in COVID-19 patients (11) (summarized in Table 1), and will likely be tested in future asthma research.

## Reevaluation of ECP as a marker for eosinophils in extracellular trap formation

We wish to raise concerns regarding the study by Lu et al. (12), which identifies eosinophil extracellular traps (EETs) as a key mechanism in allergic asthma pathophysiology. The study relies on eosinophil cationic protein (ECP) as a marker for eosinophils in extracellular trap (ET) identification. However, emerging evidence challenges the specificity of ECP as an eosinophil marker. This oversight may affect the study’s conclusions and has broader implications for understanding the mechanisms underlying allergic asthma.

## Specificity of ECP as an eosinophil marker

ECP, a cationic protein stored in eosinophil granules, has long been associated with eosinophil activity (13). These cells play a predominant role in inflammatory disorders such as asthma, where quantification of eosinophil proteins, including ECP, is commonly used to assess disease activity (13). Asthma, a chronic airway disease affecting over 300 million people globally (14), often involves eosinophilic infiltration as a hallmark of inflammation. Elevated levels of ECP in sputum or bronchoalveolar lavage fluid (BALF) from asthmatic patients have traditionally been interpreted as evidence of eosinophilic activity (15, 16). However, immunocytochemical analyses of such samples indicate that the number of ECP-positive (ECP^+^) cells often exceeds the number of eosinophils, suggesting that other cell types may also contribute to ECP release in the airways (17). Recent studies have demonstrated that ECP is not exclusively produced by eosinophils. Neutrophils, monocytes, and myelomonocytic cell lines are capable of synthesizing and releasing ECP (13, 18, 19). Our group has shown that neutrophils from allergic patients—but not eosinophils—produce ECP following stimulation with surface-bound IgE or antigens (Ags) to which the patients were sensitized (19). These findings underscore the need for caution in interpreting ECP as a marker of eosinophilic activity, particularly in inflammatory diseases like allergic asthma. Accurately identifying the cellular sources of ECP is crucial for understanding its role in disease pathophysiology and for designing targeted interventions.

## Consequences of mislabeling: pathophysiological misinterpretations and therapeutic implications

ETs are intricate structures composed of DNA, histones, and cytotoxic proteins. They are essential for pathogen neutralization but, when dysregulated, contribute to tissue damage in inflammatory conditions, including asthma (20). Multiple cell types, including mast cells, eosinophils, macrofages/monocytes (21), B cells, T cells, basophils, and dendritic cells (22) have been described as sources of ETs, implicating them in the pathophysiology of asthma.

Misidentifying the cellular origin of ETs can lead to flawed conclusions about the mechanisms driving airway inflammation and hinder the development of targeted therapies. For example, therapies designed to eliminate ETs after their formation might reduce inflammation but could also impair their protective role in pathogen defense. A more promising approach involves targeting upstream signals that trigger ETs formation, thereby preventing excessive or dysregulated production. This strategy requires an accurate understanding of the cellular mechanisms driving ET formation.

Neutrophils and eosinophils both play active roles in asthma but differ in their timing and contributions to the inflammatory response. Neutrophils are the first responders, peaking within 18 hours and often predominating in severe asthma exacerbations (23). In contrast, eosinophils infiltrate tissues later, peaking around 42 hours, and are more prominent in milder or chronic cases (23). This distinction highlights the importance of distinguishing between neutrophil- and eosinophil-derived ETs in asthma research.

## Revisiting the findings of Lu et al.

A previous report demonstrated the clinical relevance of neutrophil-derived ETs in a mouse model of asthma exacerbation triggered by infections (2). However, our recent findings indicate that neutrophils, but not eosinophils, from allergic asthma patients form ETs in response to sensitizing antigens, even in the absence of infection (6). This contrasts with the findings of *Lu* et al. (12), who claimed that ETs in a mouse model of antigen-induced asthma were eosinophil-derived, based on double-positive staining for ECP and citrullinated H3-histone (cit-H3) (a recognized marker of ETs) (24).

The study by *Lu* et al. also identified ECP^+^/cit-H3^+^ structures in airway samples from pollinic asthmatic patients during pollen season, which were extrapolated as eosinophil extracellular traps (EETs) based on their murine model. However, extrapolating findings from animal models to human disease is fraught with challenges. Murine asthma models lack the neutrophil infiltration characteristic of human asthma, where neutrophils often dominate allergen-driven airway inflammation (25). Our own data show that sensitized neutrophils from allergic asthma patients form NETs that are positively stained for neutrophil elastase (NE, a neutrophil-specific marker) and ECP (6). These findings suggest that the ECP^+^/cit-H3^+^ structures observed in human samples by Lu et al. may have been misattributed to eosinophils.

Misinterpreting the cellular sources of ETs, as appears to be the case in Lu et al.’s study, could lead to misdirected therapeutic development efforts. Targeting eosinophils based on these findings might overlook the contributions of neutrophils, potentially reducing treatment efficacy.

## Recommendations for rigorous methodologies

To address these concerns, we strongly recommend the use of alternative markers with greater eosinophil specificity, such as eosinophil peroxidase (EPO) or major basic protein (MBP) (26). These markers are uniquely associated with eosinophils and would provide a more reliable assessment of eosinophil involvement in ET formation. Combining multiple markers, including both ECP and neutrophil-specific markers like CD66b, could help distinguish between NETs and EETs in complex inflammatory environments. Additionally, experimental controls should be implemented to exclude eosinophil contamination in neutrophil preparations. Advanced techniques such as flow cytometry or RNA sequencing can validate cell-specific marker expression and ensure robust conclusions. Moreover, it is imperative to incorporate clinically relevant asthma models that better reflect the cellular dynamics of human disease. The absence of neutrophil infiltration in murine models (25) limits the translational applicability of findings to human asthma. Including human-derived samples, such as BALF or peripheral blood, alongside *in vitro* studies using isolated cell populations, would provide more accurate insights into the cellular mechanisms driving ET formation and inflammation.

## Broader implications for asthma research

Asthma is a heterogeneous disease with diverse phenotypes, each associated with distinct inflammatory pathways. Accurately characterizing these pathways is essential for developing targeted therapies. For example, eosinophilic asthma often responds to anti-IL-5 therapies, which reduce eosinophil counts, while neutrophilic asthma is less responsive to corticosteroids and may benefit from alternative treatments targeting neutrophil activity. Misidentifying the cellular origins of ETs could obscure these distinctions, delaying the development of phenotype-specific therapies. Furthermore, the role of ETs in other inflammatory diseases, such as chronic obstructive pulmonary disease (COPD) and rheumatoid arthritis, underscores the need for precise methodologies in studying ET formation. Lessons learned from asthma research can inform studies in these conditions, fostering a broader understanding of ET-mediated inflammation.

## Conclusion

ETs play a critical role in asthma pathophysiology and represent promising therapeutic targets. However, accurately identifying the cell types and pathways involved in ET formation is paramount. The study by Lu et al. (12) highlights the significance of ETs in asthma but misinterprets ECP as an eosinophil-specific marker, potentially conflating the contributions of neutrophils and eosinophils. Reevaluating these findings using rigorous methodologies and specific markers is essential for advancing our understanding of ET formation and its role in asthma. Only through accurate characterization of cellular mechanisms can we develop effective, targeted therapies for this complex disease.

## Data Availability

The original contributions presented in the study are included in the article/supplementary material. Further inquiries can be directed to the corresponding author.
